# Quantification of Epigenetic Aging in Public Health

**DOI:** 10.1146/annurev-publhealth-060222-015657

**Published:** 2024-12-16

**Authors:** Cynthia D.J. Kusters, Steve Horvath

**Affiliations:** 1 Department of Epidemiology, Fielding School of Public Health, University of California, Los Angeles, California, USA; 2 Department of Biostatistics, Fielding School of Public Health, University of California, Los Angeles, California, USA; 3 Altos Labs, Cambridge, United Kingdom

**Keywords:** epigenetic age, epigenetic clock, human studies, aging

## Abstract

Estimators of biological age hold promise for use in preventive medicine, for early detection of chronic conditions, and for monitoring the effectiveness of interventions aimed at improving population health. Among the promising biomarkers in this field are DNA methylation–based biomarkers, commonly referred to as epigenetic clocks. This review provides a survey of these clocks, with an emphasis on second-generation clocks that predict human morbidity and mortality. It explores the validity of epigenetic clocks when considering factors such as race, sex differences, lifestyle, and environmental influences. Furthermore, the review addresses the current challenges and limitations in this research area.

## INTRODUCTION

In the field of public health, accurately measuring aging is increasingly important. This pursuit is driven by the need to understand the factors that contribute to aging and to identify effective interventions that extend the healthy lifespan. Researchers historically relied on telomere length as a biological indicator of aging. However, recent studies have shifted to focus on a broader array of aging hallmarks.

The year 2013 was notable for the publication of not only the pan-tissue epigenetic clock but also a highly influential review article on the hallmarks of aging (99). These hallmarks were defined as molecular and cellular processes that are associated with changes across the lifespan and have the possibility to increase or decrease with treatment, interventions, or lifestyle changes (99). The hallmarks included genomic instability, telomere attrition, epigenetic alterations, loss of proteostasis, deregulated nutrient sensing, mitochondrial dysfunction, cellular senescence, stem cell exhaustion, and altered intercellular communication. In 2023, the hallmarks of aging were updated to include impaired macroautophagy, chronic inflammation, and dysbiosis (100). The same year saw the publication of the pan mammalian methylation clock that measures age in all mammalian species (102).

In this review, we focus on DNA methylation–based biomarkers of aging (epigenetic clocks), as they provide the most precise aging estimators across humans and other mammalian species (102). However, these measures of epigenetic aging likely capture only a portion of the overall biological aging process. The twelve hallmarks are not independent and are highly correlated with each other. Indeed, in vitro studies have revealed that epigenetic clocks are linked to nutrient sensing, mitochondrial activity, and stem cell composition but seem to be disconnected from cellular senescence, telomere attrition, and genomic instability (72). Thus, even though this review discusses epigenetic alterations, specifically DNA methylation, it is important to account for the other hallmarks. Epigenetic aging, an indicator of biological age, may provide a nuanced perspective of aging and has the potential to be used as a proxy for future risks for mortality and morbidity, to determine potential risk and aging consequences of various exposures, and to identify the impact of an intervention on someone’s risk profile.

## DNA METHYLATION AND EPIGENETIC CLOCKS

DNA methylation of cytosine [5′ methylcytosine (5mC)] is the most common DNA modification and occurs mainly at cytosines in a CpG dinucleotide context in differentiated mammalian cells. The stability of 5mC in biological samples, including DNA stored over long periods, allows for extensive data collection for subsequent high-throughput analysis.

Over the past decade, we have made striking discoveries about the potential of epigenetic shifts to determine a person’s age. Epigenetics involves the chemical adjustments and structuring of the genome, which can impact or signify its activity. The effects of age on global methylation levels were first noted by Berdyshev et al. in 1967, as reviewed in Horvath & Raj (67). However, high-fidelity platforms that measure DNA methylation at the single nucleotide level were needed to develop highly accurate age prediction models that deserved the label “clocks.” These DNA methylation–based age estimators have revolutionized how we measure age across all tissues and nucleated cells by tracking the abundance of molecules (methyl groups) that attach to DNA cytosines (11, 56, 61, 65, 153).

We define epigenetic clock as a multivariate age (or mortality risk) predictor, based on a multivariate regression model involving multiple cytosines. The first epigenetic clock, developed in 2011, estimated age from human saliva samples (11), followed by the pan-tissue clock in 2013, which applied to all human DNA sources except sperm (61). Other significant tissue-specific estimators include Hannum and colleagues’ (56) blood sample clock and various sparse clocks based on a few CpGs (65, 91, 103, 153). We group the many different epigenetic clocks by generation.

## GENERATIONS OF EPIGENETIC CLOCKS

The classification into first-, second-, and third-generation epigenetic clocks generally refers to the progression in the development and utility of these clocks.

### First-Generation Epigenetic Clocks

First-generation clocks are typically designed to predict chronological age accurately across a wide range of tissues and cell types. These include the original Hannum clock (56). Another example is the Horvath pan-tissue clock, which uses 353 CpG sites (regions of DNA where a cytosine nucleotide is followed by a guanine nucleotide) to predict chronological age (61). While these clocks can reflect biological age and are associated with a variety of age-related conditions, they were not initially designed to predict health outcomes. Even though research on first-generation clocks continues using novel approaches, including deep learning (31, 46, 124), the most precise clocks may not lead to significant biological insights (163). These clocks have high correlations (*r*) with chronological age, with the majority above 0.9 all the way up to 0.99 (150). However, even with high correlations, the median absolute error (MAE) is high at 3.8 years, which suggests that while these metrics are valuable for population-based studies, they may not be accurate enough for personalized medicine (34).

### Second-Generation Epigenetic Clocks

Second-generation clocks were developed to predict not just chronological age but also health span and lifespan and thus are often associated with age-related morbidity and mortality. The two most familiar clocks in this category are the PhenoAge and GrimAge clocks (91, 103). GrimAge is especially well suited to predict time-to-death, time-to–coronary heart disease, and time-to-cancer, with hazard ratios for mortality between 1.1 and 1.8 among various studies (59, 101, 109, 113). The GrimAge clock was built using several DNA methylation–based surrogate measures for plasma proteins, including leptin and plasminogen activator inhibitor-1, and smoking pack-years (103). Lu et al. (101) recently developed an updated version of GrimAge that also includes DNA methylation–based estimates of c-reactive protein and hemoglobin A1C, which slightly enhances the association with morbidity. The updated version outperforms the earlier version across racial and ethnic groups, with a hazard ratio between 1.06 and 1.15 for mortality and between 1.06 and 1.21 for coronary heart disease (101).

Researchers developed two epigenetic DNA methylation–based biomarkers that do not estimate age per se but estimate a rate of aging instead: Dunedin POAM (pace of aging methylation) and PACE (6, 7). The outcome, pace of aging, was estimated based on health data, such as body mass index, HbA1c, grip strength, lung function, cognitive decline, and subjective health status following individuals over a 20-year period (6, 7). Individuals with a PACE rate faster than 1 standard deviation (SD) or more above the mean, compared with those with an average rate (within 1 SD of the mean), had a hazard ratio of 1.26 [95% confidence interval (CI): 1.14–1.40] for mortality and a hazard ratio of 1.16 (95% CI: 1.12–1.20) for chronic disease morbidity (7).

A recent longitudinal study among European Caucasians validated the use of epigenetic clocks with mortality risks, when reviewing both epigenetic age at baseline and change of epigenetic age over time (84). The most accurate prediction for mortality was for the PhenoAge and GrimAge clocks with a hazard ratio of 1.31 (95% CI: 1.17–1.46) per SD for baseline and 1.23 (95% CI: 1.10–1.37) for the slope for the PhenoAge as well as a hazard ratio of 1.46 (95% CI: 1.29–1.65) per SD for baseline and 1.13 (95% CI: 1.02–1.26) for the slope for the GrimAge clock (84). This study indicates that both baseline epigenetic age and changes in epigenetic age over time independently predict mortality, implying that interventions that lower epigenetic age may reflect changes in mortality risk.

### Third-Generation Epigenetic Clocks

Third-generation clocks utilize cytosine methylation levels within highly conserved DNA segments to determine chronological age across multiple species. Thus, a single regression model based on the same covariates (cytosines in conserved stretches of DNA) is used to predict age across multiple species (63, 102, 145). The resulting multispecies clocks hold the potential to bridge the gap between animal model research and human applications, thus facilitating the translation of research findings. This work is especially important for ongoing animal intervention studies, as successful interventions may not translate in humans. Several pan-mammalian clocks have been developed, including a mammalian clock for estimating relative age, which is defined as the ratio between age and maximum lifespan (102). The pan-mammalian clock is also useful for predicting human mortality risk in epidemiological cohort studies. Furthermore, conditions such as obesity and inflammation, measured by c-reactive protein, appear to accelerate aging according to the pan-mammalian clock, whereas vegetable intake seems to slow it down (102). Currently, second-generation clocks, such as GrimAge, are significantly more accurate in predicting human mortality risk and associated factors compared with the pan-mammalian clock (102), whereas third-generation clocks are more useful for translating animal-to-human studies aimed at interventions that influence the aging process.

## FUTURE OF EPIGENETIC CLOCKS

Over the last few years, many new types of epigenetic clocks have been developed. Not merely chronological age trackers, these clocks have been instrumental in revealing associations between accelerated aging and numerous health aspects through analysis of diverse human DNA methylation profiles (67).

The most recent epigenetic clocks can be divided into new epigenetic clocks using novel modeling techniques or more complicated methodology (39, 46, 108) developed for a specific subpopulation (e.g., for children or centenarians) (12, 33, 52, 78, 111) or for different tissue, including placental tissue (88, 129), and saliva/buccal cells (35, 111, 133).

Although the second-generation epigenetic clocks are correlated with each other and are all associated with life- and health span, each individual clock was developed on its own training dataset and may be associated with slightly different health-related outcomes. When an exposure or outcome is associated with multiple clocks, the association is robust. In contrast, when associations are identified among only a subgroup of clocks, this finding may indicate the presence of different underlying mechanisms or could be due to spurious findings (119, 130).

In the following discussion, we present a curated selection of significant observations concerning epigenetic aging and its implications for public health rather than offering an exhaustive compilation of all available epigenetic clocks. Throughout most of this review, our attention is directed toward the three second-generation epigenetic clocks that are predominantly utilized at the time of writing. We exclude studies that lie outside the realm of second-generation clocks because first-generation clocks were covered in several previous reviews (5, 40, 67).

## UNDERLYING MECHANISMS PROPOSED WITH THE EPIGENETIC CLOCKS

Age-related gain of methylation can be observed at polycomb repressive complex 2 (PRC)-binding sites in all mammalian species, highlighting the intricate relationship between fundamental developmental pathways and aging mechanisms (102). Moqri et al. (115) recently developed clocks that focus exclusively on methylation levels at PRC2 target sites.

Many epigenetic clock studies provide substantial backing for Barker’s hypothesis concerning the developmental origins of chronic diseases (105). The epigenetic clock theory of aging posits that DNA methylation patterns provide a continuous readout of age throughout the entire lifetime, linking purposeful processes during development and cancer protection to later adverse effects causing organ dysfunction (67). Developmental aging theories emphasize how intentional and deterministic developmental processes can lead to organ dysfunction as time progresses (within the framework of antagonistic pleiotropy) (67). de Magalhães (32) recently proposed the analogy that aging could arise from a developmental software error. Overall, extensive research indicates that developmental programming plays a significant role in chronic diseases, promising an unexpected interpretation of aging and its related dysfunctions.

Each epigenetic clock is based on a distinct set of CpG sites and effect estimates. These associations are frequently agnostic to biology and derived through a mathematical optimization procedure (such as penalized regression). However, since each clock is constructed using a particular population and diverse aging groups (clocks for pediatric patients versus those for centenarians), the underlying mechanisms may differ among specific epigenetic clocks. Furthermore, while clocks serve as proxies for aging, it remains uncertain which underlying CpGs are causal or simply reflect aging processes. One way to identify the potential causal effects of clocks and aging is through Mendelian randomization (MR) analyses. MR has been used to select CpGs that have a causal effect on age-related conditions (159). This ambiguity underscores the complexity of interpreting epigenetic clock data and emphasizes the need for further research to elucidate their underlying mechanisms and implications for aging (67).

### Various Tissues Used in Clocks

Most epigenetic clocks were developed using blood samples, since blood samples are often available from previously established cohort studies (83, 120, 144). However, blood is not always the tissue of interest, e.g., when reviewing whether certain diseases are associated with an increased epigenetic age. While this discrepancy in tissue is a caveat of the most commonly used clocks, investigators have described a modest to moderate correlation between blood and brain and between blood and muscle tissue (53, 98, 135, 141). Other studies have indicated that certain tissues tend to measure older or younger than anticipated using first-generation clocks; e.g., breast tissue tends to age faster, whereas the cerebellum appears to age more slowly (56, 61, 64). However, aging may vary across tissues within an individual, indicating heterogeneity of systems aging. To address this issue, one can use tissue-specific epigenetic clocks, e.g., the cortical clock (132) or the skeletal muscle clock (147).

While blood samples are commonly used in observational studies of adults, collecting blood can be challenging in pediatric cases or very elderly individuals. Consequently, several epigenetic clocks have been developed using saliva or buccal cells (70).

### Specific Age Categories

Specialized epigenetic clocks are emerging to cater to specific subpopulations. Notably, clocks have been devised for pediatric and centenarian cohorts to address the extremes of the lifespan. Epigenetic age tends to be lower among the older population. Centenarians or supercentenarians can have 5–20 years lower age acceleration (1, 28, 30, 36, 54, 66, 80), possibly indicating either slower aging leading to their longevity or the influence of training data sourced primarily from the adult lifespan (typically between 18 and 80 years) (151).

Dec et al. (33) devised a clock tailored specifically for centenarians and conducted an epigenome-wide association study (EWAS) to explore the aging process, stratified by age category. This analysis revealed nuanced differences in aging processes between centenarians and younger individuals, suggesting that previously developed epigenetic clocks might not accurately capture epigenetic age in this older demographic. This centenarian clock estimates primarily chronological age and thus is categorized as a first-generation clock. Consequently, while it may not be as effective at predicting mortality and morbidity risk compared with second-generation clocks, it serves a valuable role in validating claims of exceptional old age.

In addition to the centenarian clocks for the end of the lifespan, several clocks have been developed for the early part of the lifecycle. There are currently four clocks to decipher gestational age: two based on blood tissue (e.g., cord blood) (12, 78) and two based on placenta tissue (88, 107). Placental age has been associated with early-onset preeclampsia and prenatal depression, suggesting that placental aging may be part of the underlying mechanism (107, 129). One clock, the NeoAge, was developed to determine neonatal aging in preterm infants; it uses buccal cells and can estimate the gestational (postmenstrual) age and the postnatal age (52). For children, the best epigenetic clock at this point is the pedBE clock, based on buccal cells, which has a strong correlation (*r* = 0.98; MAE 0.35 years) with chronological age (111). PedBE has been associated with maternal exposure during pregnancy, suggesting that even in-utero contact with harmful exposures or lifestyle could influence the aging process of the offspring (43, 140). Early exposures, such as early-life adversity, have been associated with increases in PedBE, which is subsequently associated with long-term neurodevelopmental effects in later stages of childhood (48, 134). One study has shown that pedBE can be reduced through positive parenting intervention in children with developmental delays (142), suggesting that PedBE may serve as both an indicator of success and an indicator of high-risk children for future health interventions.

### Sex Differences

According to a study by Horvath et al. (62) in 2016, men typically exhibit an older epigenetic age compared with women when blood samples are evaluated. This finding was confirmed in a recent study using second-generation clocks that used samples worldwide and a diverse sample in the United States (27, 160). In addition to these findings, a recent study indicated sex differences with several epigenetic clocks. The study showed specific patterns that varied by sex; e.g., among females, GrimAge and GrimAge2 were associated with obesity and depression (increase of 0.9–1.9 years in age acceleration among females versus 0.4–0.7 years among males), whereas in males, stronger associations were identified with hypertension, diabetes, and chronic kidney disease (increase of 0.9–3.0 years among males versus 0.2–0.8 years among females) (123). Although cross-sectional studies cannot determine directionality, they may indicate differences in underlying mechanisms or risk profiles that could explain variations in prevalence. These differences could also be attributed to variations in hormone profiles between men and women.

### Influence by Hormones

Gender differences underscore the potential link between hormone levels and epigenetic aging. Our understanding of the relationship between hormone levels and epigenetic clocks is still in its infancy, making it challenging to establish broad principles regarding their interaction. However, given the fundamental role of hormones in human development and bodily maintenance, they may significantly influence the pace and progression of the epigenetic clock.

One of the areas where this relationship becomes evident is during puberty. The rate of pubertal development in girls has been connected to their epigenetic age (10) and epigenetic age acceleration at about age 40 (55). This notion suggests that the hormonal changes occurring during this critical developmental stage may have a substantial impact on an individual’s biological aging process and could highlight a critical window of time for interventions.

Menopause, a significant hormonal transition in women, is another stage that appears to have a profound impact on the epigenetic clock. Early onset of menopause is associated with an increase in epigenetic age (90). This observation suggests that the decline in estrogen levels characteristic of menopause may accelerate biological aging, but further research is needed to confirm this hypothesis. Conversely, menopausal hormone therapy is associated with a lower epigenetic age in buccal cells but not in blood (90). A recent cohort study indicated that hormone therapy among postmenopausal women was associated with a decrease in phenotypic age, with larger beneficial effects among women with lower socioeconomic status (SES), though overall effects were very minimal (96). Although this study did not use epigenetic age, phenotypic age is strongly correlated with epigenetic PhenoAge (*r* = 0.74) (90), suggesting that similar associations would likely be observed for PhenoAge. These studies highlight that hormone therapy may have beneficial implications on mortality and morbidity and on how epigenetic age can be used as a proxy for the efficacy of an intervention.

In men, a correlation has been observed between testosterone levels and epigenetic clocks (86). This association implies that variations in testosterone, a hormone integral to male physiology, and testosterone–estradiol ratio, an indicator for hormonal balance (androgens/estrogens), might influence the pace of the epigenetic aging process. However, the nature and extent of this relationship require more extensive investigation.

In addition to sex hormones, hormones related to the somatotropic axis have been proposed to influence biological aging. The somatotropic axis is a regulatory system that includes hormones such as growth hormone and insulin-like growth factors. Several studies have found increased longevity in animal models with growth hormone receptor knockouts. Disruptions of the somatotropic axis lead to decreased epigenetic age in mouse models. Mice lacking growth hormone production (Snell mice) or growth hormone receptor knockout mice (GHRKO mice) exhibit lower epigenetic ages according to most mouse clocks, including the pan-mammalian clock (101).

Some recent studies have found that metformin, a well-known medication for diabetes that improves insulin sensitivity and lowers glucose levels, has been associated with a decrease in epigenetic age among monkeys (157). In addition, two small observational studies indicated that metformin, or metformin in combination with growth hormone and dehydroepiandrosterone, was associated with lower and decreasing epigenetic age by around two years, respectively (38, 93). As such, a phase II trial is currently recruiting to assess whether metformin does improve methylation age among males as well as other indicators for senescence. Overall, these novel studies indicate that there may be interventions related to hormones that could protect individuals from biological aging and improve mortality/morbidity risks. However, how much these hormones could protect, or whether specific subgroups, such as males only, would benefit more from treatment, still needs to be established.

## VALIDATION AMONG EPIGENETIC CLOCKS AMONG ETHNIC POPULATIONS

Several studies have validated these clocks across ethnically diverse populations. For instance, both PhenoAge and GrimAge have been validated to be associated with mortality and age-related morbidity in US populations of different ancestries, including Hispanic, African, and European ancestry. Another example is a Taiwanese study that used second-generation clocks (95). Furthermore, these clocks were validated in most European populations such as an Irish longitudinal population, demonstrating associations with mortality and age-related outcomes (109). While Dunedin PACE was originally developed on white New Zealanders, subsequent studies have observed changes in Dunedin PACE across diverse ethnic backgrounds, including Han Chinese, Native Americans, African Americans, and Hispanics (114). In addition, GrimAge2 was developed on an admixed population and subsequently validated across various ethnic groups, including White non-Hispanics, African Americans, and Hispanics (101).

## DIFFERENCES OF EPIGENETIC AGING AMONG VARIOUS POPULATIONS

Variations in epigenetic aging among different ethnic populations could stem from limitations inherent in the development of epigenetic clocks. Nevertheless, the main second-generation clocks are now applied across diverse populations and have undergone validation. Discrepancies have indeed been observed when comparing ethnic groups (49, 62, 97, 160). One large meta-analysis studied Pheno-, GrimAge, and Dunedin PACE across 22 countries and 31 ethnicities, drawing data from the Gene Expression Omnibus. Individuals were derived from all continents, with the largest populations from the United Kingdom and United States (160). This study indicated that Chinese and Malays exhibit accelerated aging compared with Indians, while White Americans age marginally slower than do African Americans, Latinos, and Asian Americans. Yakuts age at a quicker rate than do Russians, and among all the populations considered, Congolese, Malays, and Tsimanés demonstrate the most rapid aging (160). These distinctions are not attributed solely to ethnic background but also reflect geographical and regional disparities. In broad terms, White non-Hispanic individuals appear to age more slowly. The observed increases in epigenetic age among minorities are theorized to result from diverse environmental and psychosocial exposures. To assess whether certain populations may be at higher risk for accelerated biological aging, we need to examine the impact of environmental and psychosocial factors in relation to their genetic imprint in order to identify modifiable risk factors that could be targeted to reduce biological aging disparities.

## ENVIRONMENTAL AND PSYCHOSOCIAL CHARACTERISTICS AND EPIGENETIC AGING

Research on some of these environmental and psychosocial factors is ongoing and rapidly expanding, highlighting how variables such as socioeconomic factors, psychosocial stress, trauma, environmental exposures, and lifestyle can influence epigenetic aging. This growing body of evidence underscores the importance of how these factors may influence aging and also who in the general population may be at an increased risk for developing aging-related morbidity or is at a higher risk for premature death.

### Socioeconomic Factors

Several studies have identified that poverty is associated with an increased epigenetic age (2, 19, 24, 29, 110, 131, 143). Economic hardship during adolescence during the Great Recession has been associated with an average of a 1.4-year increase in Hannum and Horvath age among African Americans several years later (postrecession) (24). Several studies have indicated that this finding may be different for specific subgroups within their populations. However, results are not consistent and do not indicate a specific high-risk subpopulation. For example, in the Healthy Aging in Neighborhoods of Diversity across the Life Span (HANDLS) cohort, poverty was associated with an increased Dunedin PACE, with a 7% increase in aging rate. This effect was stronger among populations of European ancestry (12% increased rate versus 5% among those with and without childhood poverty), whereas in individuals who identified as African American, there was no increase in PACE related to childhood poverty status (10% versus 10%); however, Dunedin PACE was significantly higher among the African American population above the poverty line compared with those of European ancestry above the poverty line (131). This finding may suggest that other socioeconomic factors, but not poverty, are associated with an increased epigenetic age among African Americans (110). Assari & Zare (2) identified an effect of childhood poverty on PhenoAge at age 15 (−0.09 years per income-to-poverty ratio), with a pronounced effect among males (−0.13 years), but a reduced and not statistically significantly effect was observed among females (−0.06). A study discovered that the impact of childhood poverty on epigenetic age acceleration was partially mediated by lifetime smoking (48–80%). Although these results are promising, mediation was measured at the same time as the outcome. In addition, the underlying pathways may be more complex than a direct mediation pathway (110). Even after adjusting for age, race/ethnicity, sex, several poverty characteristics, marital status, education, and lifestyle (e.g., alcohol, smoking), food insecurity was associated with an increased GrimAge, as well as an increased second-generation Zhang clock (143).

Neighborhood deprivation or SES has also been associated with increased epigenetic age (average of a 0.3–0.8-year increase in epigenetic age acceleration) (71, 87, 89, 106, 128, 136, 137). One study found that this association (*r* = −0.09 among total population with Dunedin PACE) was slightly reversed among individuals who reported physical activity or a healthy eating index, suggesting that individual-level behavior may protect from biological aging. Unfortunately, the effects of this positive behavior were very minimal and appeared to differ by ethnic background (106).

Lower education is a well-known risk for higher epigenetic age, potentially mediated through unhealthy lifestyles (27, 42, 81, 127). One large, multicohort study indicated that educational attainment was associated with GrimAge acceleration (34 excess deaths per 10,000 person-years for those with education inequalities) and mortality (57 excess deaths per 10,000 person-years) and that an increase in GrimAge acceleration was mediating the increase in mortality by 60%. However, this effect was seen only among males, whereas the effect of educational attainment was minimal on both GrimAge and mortality (42). However, another study indicated that upward mobility in education across multiple generations and social mobility from childhood to later life was associated with lower Dunedin PACE within the Framingham Heart Study cohort (50, 51), though results were not consistent with findings in the Health and Retirement Study, which did not find any evidence of upward mobility and epigenetic aging (81).

As more advanced and better-powered studies emerge, we will gain extensive knowledge of how poverty, neighborhood exposure, and education contribute to accelerated aging and identify those at highest risk for negative consequences. However, the complexity of interactions between various socioeconomic variables, along with inconsistencies across studies in identifying who is most at risk and how these associations are mediated by modifiable factors, makes it challenging to determine the best public policy course of action. Nevertheless, continued research will provide valuable insight that could guide targeted interventions and the identification of high-risk populations.

### Psychosocial Stress and Trauma

The relationship between psychosocial stress and accelerated epigenetic aging has been explored in several review papers, highlighting a consistent link between chronic stress and increased epigenetic age across multiple studies (45, 60, 104, 121, 125, 138, 161). For example, in the Women’s Health Initiative, stressful life events were linked to a 0.34-year increase in GrimAge acceleration (138). Similarly, perceived stress at age 11 was associated with a 0.14-year increase in GrimAge at age 40 (89). Cumulative stress was associated with epigenetic aging by 0.48 years, which was modulated by emotion regulation and self-control (57). In addition, several other studies identified that resilience may buffer the effect of stress on epigenetic age acceleration (8, 162).

Perceived (racial) discrimination has been associated with an increased GrimAge among various racial groups, even after adjusting for income, education, and neighborhood disadvantage, with an average epigenetic age acceleration between 0.4 and 1.8 years (99, 106–108). In the Health and Retirement Study, perceived discrimination was associated with increases in various epigenetic clocks, including GrimAge and DunedinPoAm. Depressive symptoms partially mediated this increase by 20–50%, highlighting the potential need to address depressive symptoms in order to mitigate the impact of discrimination on biological aging (9). While perceived racial discrimination has been associated with an increase in Hannum age [0.62 years in the Strong African American Healthy Adult Project (SHAPE) and 1.45 years in the Adults in the Making (AIM) study] among African American youth, supportive family appears to mediate this association (−0.54 years for SHAPE; −0.43 years for AIMS for the interaction between discrimination and family support). Although these findings are very promising and were consistent across two study populations, further studies need to validate these findings in a longitudinal study (18). This result aligns with the hypothesis that even in adverse environments, social support and resilience can partially mediate negative effects.

Traumatic events, such as adverse childhood experiences (ACEs), also have detrimental effects on epigenetic aging. Multiple studies have reported that ACEs are associated with an increase in second-generation clocks among young adults (6, 7, 14, 55, 58, 74). In addition, both lifetime trauma and post-traumatic stress disorder (PTSD) have been linked to accelerated epigenetic aging (14, 60, 73, 94, 156). Emerging evidence suggests that individual coping styles and resilience may partially mediate the relationship between trauma and epigenetic age acceleration (8). However, the relationship between resilience and trauma is complex, with some studies indicating that this relationship requires further in-depth investigation (112, 118).

In conclusion, there is consistent research supporting a link between psychosocial stress, discrimination, and trauma and a small increase in epigenetic age. Resilience and social support are emerging as potential moderators. However, future studies, particularly longitudinal studies and those with more complex modeling, are essential to further clarify these relationships and their implications for mitigating biological aging.

### Environmental Toxins

Research on the impact of air pollution on epigenetic age is limited and challenging due to methodological constraints. While some studies suggest a link between higher air pollution levels and an increase in second-generation epigenetic age, inconsistencies exist, and pinpointing the exact causative exposures remains difficult (79, 139, 154). For instance, one study found that among non-Hispanic White women, certain clusters of PM_2.5_, possibly from sources such as wood smoke and surface oil, were associated with a six-year increase in PhenoAge per interquartile range (154). In addition, several studies suggest that these effects are more pronounced among Black participants, possibly due to their higher exposure levels (79). Exposure to metals, including arsenic, was associated with an increased epigenetic age (0.4-year, 0.8-year, and 0.01-year increase in PhenoAge, GrimAge, and Dunedin PACE, respectively) among a Native American community (15, 69). Arsenic gestational exposure was also linked to epigenetic age at birth, while prenatal and early-life arsenic exposures were suggestively associated with PhenoAge among Chilean adults (16, 17).

### Lifestyle Characteristics

While psychosocial stress and environmental exposures appear to increase epigenetic aging, this outcome may be moderated by a healthy lifestyle. Physical activity is associated with a decrease in aging-related morbidity and mortality, whereas the effect on epigenetic age seems more limited. For example, the effects of physical activity on GrimAge acceleration were null among the Coronary Artery Risk Development in Young Adults (CARDIA) study participants at age 20 (75), whereas other studies indicated that physical activity was associated with slower epigenetic aging, potentially due to changes in immune cell composition and a decrease in cardiovascular risks (3, 41, 44, 75, 117, 126).

Previous studies have indicated that a healthy diet with higher intake levels of fruits, vegetables, and fish or a Mediterranean diet is associated with a lower epigenetic age, indicating a slightly younger biological age (47, 76, 82, 92, 126, 146, 149). These effects may already be present in utero, as one study found that maternal intake of more fat was associated with increased epigenetic age acceleration by six weeks among newborns, whereas vitamin D supplementation may decrease epigenetic age by eight weeks (122).

In addition, several studies in humans, similar to animal studies, have reviewed calorie restriction as a potential means to increase lifespan using epigenetic clocks as a proxy for change in biological age and effect of intervention on the risk profile for decreased mortality. Caloric restriction decreased the Dunedin PACE in the Comprehensive Assessment of Long-Term Effects of Reducing Intake of Energy (CALERIE) study, with effects still visible after 2 years by ~0.02 years, though no effects were found for the PC GrimAge or PC PhenoAge (152).

Alcohol is well known for a J-shape association with mortality, where the use of alcohol in limited portions has the lowest mortality rate. Alcohol consumption has been associated with a slight increase in epigenetic age [0.45 year increase in GrimAge per ln(drinks/week) among African Americans and between 0.4 and 0.8 years in PhenoAge/GrimAge acceleration per drink in the Framingham Heart Study] (13, 148, 164). The effect may be stronger when using an epigenetic proxy instead of socially desirable answers from a questionnaire (4).

Smoking is a well-known risk for various comorbidities and mortality and, as such, is also very strongly associated with epigenetic aging among diverse populations (20, 23, 27, 77, 101, 103, 164). It is important to highlight that GrimAge and GrimAge2 both use an epigenetic proxy for smoking pack-years as a component within the clock because it is strongly associated with health span and lifespan (101, 103). While smoking itself is associated with an increase in epigenetic age, smoking cessation can reverse this effect. One study found that three months after smoking cessation, epigenetic age acceleration within the airway cells was similar to that among nonsmokers (155).

Healthy sleep has consistently been associated with lower epigenetic age (142–145). To date, only a few studies have examined the effects of insomnia symptoms and short sleep on epigenetic aging. These studies have indicated increased epigenetic aging among women postpartum and postmenopausal with insomnia symptoms and sleep deprivation and among an older population in the Health and Retirement Study in the United States (21, 22, 85). These studies could indicate that short sleep duration causes an accelerated aging process (3, 41, 44, 75, 117).

## INTERVENTION AND HUMAN CLINICAL TRIALS

Given the effects of lifestyle on epigenetic age in cross-sectional studies, researchers have conducted several longitudinal studies on lifestyle interventions. Epigenetic clocks have been employed in several phase 1 clinical trials. While the primary objective of these trials was to demonstrate safety rather than effectiveness, there were indications of a possible epigenetic rejuvenation effect, as noted in studies by Fahy et al. (38) and Clement et al. (26). Lifestyle interventions to slow aging, resulting in a reduced epigenetic age, include caloric restriction and exercise. In addition to lifestyle interventions, use of medications such as metformin and rapamycin has been proposed (116).

One small study among females indicated a decrease in GrimAge after women were assigned a low-glycemic, high-antioxidant, and mainly plant-based diet but found no effects for physical activity (41). A healthy Mediterranean diet with high polyphenols has been associated with a decrease in epigenetic age after 18 months (158).

Caloric restriction has shown strong effects on longevity in animal studies. However, feasibility of restricting calories among humans is limited, and it would be important to recognize that long-lasting restriction could also have negative effects. The CALERIE study randomly assigned adults to a 25% caloric restriction with no effects on PhenoAge or GrimAge but a modest effect on PACE. Dunedin PACE was decreased even after two years of the intervention; however, no measurements were performed after the intervention ended (152). While calorie restriction is a very promising intervention, it may be difficult to implement in the real world.

In addition to lifestyle and medication, plasmapheresis has been proposed to have rejuvenation effects and has had promising results in animal studies (25, 37, 68). So far, one small study in humans indicated that plasma transfusion with human umbilical cord plasma injected weekly for 10 weeks decreased GrimAge by an average of 0.8 years (26).

While these intervention studies are promising, more research needs to be performed. The effect estimates appear limited, and some of these interventions are not feasible to implement for a longer duration or on a larger population. The studies that have been performed so far were done on a small sample size, with limited ethnic diversity, and the duration of these effects postintervention has yet to be determined.

## THE FUTURE OF EPIGENETIC CLOCKS IN PUBLIC HEALTH RESEARCH

Hundreds of studies have demonstrated the relevance of epigenetic clocks to disease risk factors. These clocks have shown significant associations with numerous lifestyle factors such as diet, exercise, smoking, and stress, all of which play pivotal roles in determining the risk of various diseases. By providing a measure of biological age that reflects the cumulative impact of these factors, epigenetic clocks can offer a more nuanced understanding of disease risk. In future public health research, investigators must carefully consider the DNA source and recognize that a difference in epigenetic age acceleration, unrelated to the exposure of interest (e.g., confounding by genetic ancestry and sex), may exist within the study populations. In addition, while DNA derived from blood samples is commonly utilized, it may not fully capture tissue-specific effects. Therefore, it is important to exercise caution when interpreting epigenetic age acceleration as a proxy for diseases related to specific tissues.

Epigenetic clocks have shown predictive power for certain age-related conditions, including metabolic syndrome, cardiovascular disease, atrial fibrillation, kidney disease, and even certain types of cancer. In some instances, accelerated epigenetic aging precedes the onset of these diseases, highlighting their potential for early detection and prevention in large populations. However, further development is needed to determine if improved epigenetic clocks can achieve the accuracy required for individual health monitoring and personalized health care. Improved future versions of epigenetic clocks could transform our approach to disease prevention and public health promotion.

While current epigenetic clocks are not yet accurate enough for personalized medicine, they can help identify subpopulations at higher risk following a specific exposure, underscoring the potential for targeted interventions. In addition, these clocks are valuable for assessing whether an intervention or policy change may impact long-term health outcomes and which subgroup it affects most. However, to date, few studies have fully harnessed this potential in public health practice. This area of research remains underexplored and warrants further investigation and application.

## CONCLUSION

Epigenetic clocks, which estimate biological age through patterns of DNA methylation, present an exciting prospect for public health. With the ability to provide a picture of an individual’s aging process, second-generation clocks predict mortality risk better than traditional demographic variables such as age, sex, and ethnicity do.

By identifying individuals who are aging faster biologically, interventions could be targeted more effectively, potentially slowing the aging process and leading to significant improvements in population health. Ongoing research in the area of DNA methylation–based biomarkers remains an exciting frontier in preventive medicine.

## References

[1] Armstrong NJ, Mather KA, Thalamuthu A, Wright MJ, Trollor JN, 2017. Aging, exceptional longevity and comparisons of the Hannum and Horvath epigenetic clocks. Epigenomics 9(5):689–700 28470125 10.2217/epi-2016-0179

[2] Assari S, Zare H. 2024. Poverty status at birth predicts epigenetic changes at age 15. J. Biomed. Life Sci 4(1):989 39087138 10.31586/jbls.2024.989 PMC11288982

[3] Aurich S, Müller L, Kovacs P, Keller M. 2023. Implication of DNA methylation during lifestyle mediated weight loss. Front. Endocrinol 14(Aug.):1181002 10.3389/fendo.2023.1181002 PMC10442821 37614712

[4] Beach SRH, Ong ML, Gibbons FX, Gerrard M, Lei M-K, 2022. Epigenetic and proteomic biomarkers of elevated alcohol use predict epigenetic aging and cell-type variation better than self-report. Genes 13:1888 36292773 10.3390/genes13101888 PMC9601579

[5] Bell CG, Lowe R, Adams PD, Baccarelli AA, Beck S, 2019. DNA methylation aging clocks: challenges and recommendations. Genome Biol. 20(1):249 31767039 10.1186/s13059-019-1824-y PMC6876109

[6] Belsky DW, Caspi A, Arseneault L, Baccarelli A, Corcoran DL, 2020. Quantification of the pace of biological aging in humans through a blood test, the DunedinPoAm DNA methylation algorithm. eLife 9:e54870 32367804 10.7554/eLife.54870 PMC7282814

[7] Belsky DW, Caspi A, Corcoran DL, Sugden K, Poulton R, 2022. DunedinPACE, a DNA methylation biomarker of the pace of aging. eLife 11:e73420 35029144 10.7554/eLife.73420 PMC8853656

[8] Bergquist SH, Wang D, Smith AK, Roberts DL, Moore MA. 2022. Hormetic association between perceived stress and human epigenetic aging based on resilience capacity. Biogerontology 23(5):615–27 35960459 10.1007/s10522-022-09985-8

[9] Beydoun MA, Beydoun HA, Noren Hooten N, Maldonado AI, Weiss J, 2022. Epigenetic clocks and their association with trajectories in perceived discrimination and depressive symptoms among US middle-aged and older adults. Aging 14(13):5311–44 35776531 10.18632/aging.204150 PMC9320538

[10] Binder AM, Corvalan C, Mericq V, Pereira A, Santos JL, 2018. Faster ticking rate of the epigenetic clock is associated with faster pubertal development in girls. Epigenetics 13(1):85–94 29235933 10.1080/15592294.2017.1414127 PMC5836971

[11] Bocklandt S, Lin W, Sehl ME, Sánchez FJ, Sinsheimer JS, 2011. Epigenetic predictor of age. PLOS ONE 6(6):e14821 21731603 10.1371/journal.pone.0014821 PMC3120753

[12] Bohlin J, Håberg SE, Magnus P, Reese SE, Gjessing HK, 2016. Prediction of gestational age based on genome-wide differentially methylated regions. Genome Biol. 17(1):207 27717397 10.1186/s13059-016-1063-4 PMC5054559

[13] Bøstrand SMK, Vaher K, de Nooij L, Harris MA, Cole JH, 2022. Associations between alcohol use and accelerated biological ageing. Addict. Biol 27(1):e13100 34636470 10.1111/adb.13100 PMC7614236

[14] Bourassa KJ, Caspi A, Brennan GM, Hall KS, Harrington HL, 2023. Which types of stress are associated with accelerated biological aging? Comparing perceived stress, stressful life events, childhood adversity, and posttraumatic stress disorder. Psychosom. Med 85(5):389–96 37053097 10.1097/PSY.0000000000001197 PMC10239326

[15] Boyer K, Domingo-Relloso A, Jiang E, Haack K, Goessler W, 2023. Metal mixtures and DNA methylation measures of biological aging in American Indian populations. Environ. Int 178:108064 37364305 10.1016/j.envint.2023.108064 PMC10617409

[16] Bozack AK, Boileau P, Hubbard AE, Sillé FCM, Ferreccio C, 2022. The impact of prenatal and early-life arsenic exposure on epigenetic age acceleration among adults in Northern Chile. Environ. Epigenet 8(1):dvac014 35769198 10.1093/eep/dvac014 PMC9235373

[17] Bozack AK, Rifas-Shiman SL, Baccarelli AA, Wright RO, Gold DR, 2024. Associations of prenatal one-carbon metabolism nutrients and metals with epigenetic aging biomarkers at birth and in childhood in a US cohort. Aging 16(4):3107–36 38412256 10.18632/aging.205602 PMC10929819

[18] Brody GH, Miller GE, Yu T, Beach SRH, Chen E. 2016. Supportive family environments ameliorate the link between racial discrimination and epigenetic aging: a replication across two longitudinal cohorts. Psychol. Sci 27(4):530–41 26917213 10.1177/0956797615626703 PMC4833531

[19] Brown RL, Alegria KE, Hamlat E, Tomiyama AJ, Laraia B, Crimmins EM. 2024. Psychosocial disadvantage during childhood and midlife health NIMHD social epigenomics program. JAMA Netw. Open 7(7):e2421841 39073819 10.1001/jamanetworkopen.2024.21841 PMC11287423

[20] Cardenas A, Ecker S, Fadadu RP, Huen K, Orozco A, 2022. Epigenome-wide association study and epigenetic age acceleration associated with cigarette smoking among Costa Rican adults. Sci. Rep 12(1):4277 35277542 10.1038/s41598-022-08160-w PMC8917214

[21] Carroll JE, Irwin MR, Levine M, Seeman TE, Absher D, 2017. Epigenetic aging and immune senescence in women with insomnia symptoms: findings from the Women’s Health Initiative Study. Biol. Psychiatry 81(2):136–44 27702440 10.1016/j.biopsych.2016.07.008 PMC5536960

[22] Carroll JE, Ross KM, Horvath S, Okun M, Hobel C, 2021. Postpartum sleep loss and accelerated epigenetic aging. Sleep Health 7(3):362–67 33903077 10.1016/j.sleh.2021.02.002 PMC10027398

[23] Carter A, Bares C, Lin L, Reed BG, Bowden M, 2022. Sex-specific and generational effects of alcohol and tobacco use on epigenetic age acceleration in the Michigan longitudinal study. Drug Alcohol Depend. Rep 4:100077 36285173 10.1016/j.dadr.2022.100077 PMC9592053

[24] Chen E, Miller GE, Yu T, Brody GH. 2016. The Great Recession and health risks in African American youth. Brain Behav. Immun 53:234–41 26718449 10.1016/j.bbi.2015.12.015 PMC4830489

[25] Chiavellini P, Lehmann M, Gallardo MD, Canatelli Mallat M, Pasquini DC, 2024. Young plasma rejuvenates blood DNA methylation profile, extends mean lifespan and improves physical appearance in old rats. J. Gerontol. A 79:glae071 10.1093/gerona/glae071 PMC11020299 38430547

[26] Clement J, Yan Q, Agrawal M, Coronado RE, Sturges JA, 2022. Umbilical cord plasma concentrate has beneficial effects on DNA methylation GrimAge and human clinical biomarkers. Aging Cell 21(10):e13696 36052758 10.1111/acel.13696 PMC9577957

[27] Crimmins EM, Thyagarajan B, Levine ME, Weir DR, Faul J. 2021. Associations of age, sex, race/ethnicity, and education with 13 epigenetic clocks in a nationally representative U.S. sample: the Health and Retirement Study. J. Gerontol. Ser. A Biol. Sci. Med. Sci 76(6):1117–23 33453106 10.1093/gerona/glab016 PMC8140049

[28] Cronjé HT, Nienaber-Rousseau C, Min JL, Green FR, Elliott HR, Pieters M. 2021. Comparison of DNA methylation clocks in Black South African men. Epigenomics 13(6):437–49 33677984 10.2217/epi-2020-0333

[29] Dalecka A, Polcrova AB, Pikhart H, Bobak M, Ksinan AJ. 2024. Living in poverty and accelerated biological aging: evidence from population-representative sample of U.S. adults. BMC Public Health 24:458 38350911 10.1186/s12889-024-17960-w PMC10865704

[30] Daunay A, Hardy LM, Bouyacoub Y, Sahbatou M, Touvier M, 2022. Centenarians consistently present a younger epigenetic age than their chronological age with four epigenetic clocks based on a small number of CpG sites. Aging 14(19):7718–33 36202132 10.18632/aging.204316 PMC9596211

[31] de Lima Camillo LP, Lapierre LR, Singh R. 2022. A pan-tissue DNA-methylation epigenetic clock based on deep learning. npj Aging 8(1):4

[32] de Magalhães JP. 2023. Ageing as a software design flaw. Genome Biol. 24(1):51 36973715 10.1186/s13059-023-02888-y PMC10042583

[33] Dec E, Clement J, Cheng K, Church GM, Fossel MB, 2023. Centenarian clocks: epigenetic clocks for validating claims of exceptional longevity. Geroscience 45(3):1817–35 36964402 10.1007/s11357-023-00731-7 PMC10400760

[34] Ecker S, Beck S. 2019. The epigenetic clock: a molecular crystal ball for human aging? Aging 11:833–35 30669120 10.18632/aging.101712 PMC6366965

[35] Eipel M, Mayer F, Arent T, Ferreira MRP, Birkhofer C, 2016. Epigenetic age predictions based on buccal swabs are more precise in combination with cell type-specific DNA methylation signatures. Aging 8(5):1034–44 27249102 10.18632/aging.100972 PMC4931852

[36] El Khoury LY, Gorrie-Stone T, Smart M, Hughes A, Bao Y, 2019. Systematic underestimation of the epigenetic clock and age acceleration in older subjects. Genome Biol. 20(1):283 31847916 10.1186/s13059-019-1810-4 PMC6915902

[37] Erdogan K, Ceylani T, Teker HT, Sengil AZ, Uysal F. 2023. Young plasma transfer recovers decreased sperm counts and restores epigenetics in aged testis. Exp. Gerontol 172:112042 36481396 10.1016/j.exger.2022.112042

[38] Fahy GM, Brooke RT, Watson JP, Good Z, Vasanawala SS, 2019. Reversal of epigenetic aging and immunosenescent trends in humans. Aging Cell 18(6):e13028 31496122 10.1111/acel.13028 PMC6826138

[39] Farrell C, Lapborisuth K, Hu C, Pu K, Snir S, Pellegrini M. 2021. The Epigenetic Pacemaker is a more sensitive tool than penalized regression for identifying moderators of epigenetic aging. bioRxiv. 10.1101/2021.10.05.463222

[40] Field AE, Robertson NA, Wang T, Havas A, Ideker T, Adams PD. 2018. DNA methylation clocks in aging: categories, causes, and consequences. Mol. Cell 71:882–95 30241605 10.1016/j.molcel.2018.08.008 PMC6520108

[41] Fiorito G, Caini S, Palli D, Bendinelli B, Saieva C, 2021. DNA methylation-based biomarkers of aging were slowed down in a two-year diet and physical activity intervention trial: the DAMA study. Aging Cell 20(10):e13439 34535961 10.1111/acel.13439 PMC8520727

[42] Fiorito G, Pedron S, Ochoa-Rosales C, McCrory C, Polidoro S, 2022. The role of epigenetic clocks in explaining educational inequalities in mortality: a multicohort study and meta-analysis. J Gerontol. A Biol. Sci. Med. Sci 77(9):1750–59 35172329 10.1093/gerona/glac041 PMC10310990

[43] Folger AT, Ding L, Yolton K, Ammerman RT, Ji H, 2024. Association between maternal prenatal depressive symptoms and offspring epigenetic aging at 3–5 weeks. Ann. Epidemiol 93:1–6 38479709 10.1016/j.annepidem.2024.03.001 PMC11031304

[44] Fox FAU, Liu D, Breteler MMB, Aziz NA. 2023. Physical activity is associated with slower epigenetic ageing—findings from the Rhineland study. Aging Cell 22(6):e13828 37036021 10.1111/acel.13828 PMC10265180

[45] Freni-Sterrantino A, Fiorito G, D’Errico A, Robinson O, Virtanen M, 2022. Work-related stress and well-being in association with epigenetic age acceleration: a Northern Finland Birth Cohort 1966 Study. Aging 14(3):1128–56 35113041 10.18632/aging.203872 PMC8876924

[46] Galkin F, Mamoshina P, Kochetov K, Sidorenko D, Zhavoronkov A. 2021. DeepMAge: a methylation aging clock developed with deep learning. Aging Dis. 12(5):1252–62 34341706 10.14336/AD.2020.1202 PMC8279523

[47] Gensous N, Garagnani P, Santoro A, Giuliani C, Ostan R, 2020. One-year Mediterranean diet promotes epigenetic rejuvenation with country- and sex-specific effects: a pilot study from the NU-AGE project. Geroscience 42(2):687–701 31981007 10.1007/s11357-019-00149-0 PMC7205853

[48] Gomaa N, Konwar C, Gladish N, Au-Young SH, Guo T, 2022. Association of pediatric buccal epigenetic age acceleration with adverse neonatal brain growth and neurodevelopmental outcomes among children born very preterm with a neonatal infection. JAMA Netw. Open 5(11):E2239796 36322087 10.1001/jamanetworkopen.2022.39796 PMC9631102

[49] Graf GH, Crowe CL, Kothari M, Kwon D, Manly JJ, 2022. Testing Black-White disparities in biological aging among older adults in the United States: analysis of DNA-methylation and bloodchemistry methods. Am. J. Epidemiol 191(4):613–25 34850809 10.1093/aje/kwab281 PMC9077113

[50] Graf GHJ, Aiello AE, Caspi A, Kothari M, Liu H, 2024. Educational mobility, pace of aging, and lifespan among participants in the Framingham Heart Study. JAMA Netw. Open 7(3):E240655 38427354 10.1001/jamanetworkopen.2024.0655 PMC10907927

[51] Graf GH-J, Zhang Y, Domingue BW, Harris KM, Kothari M, 2022. Social mobility and biological aging among older adults in the United States. PNAS Nexus 1(2):pgac029 35615471 10.1093/pnasnexus/pgac029 PMC9123172

[52] Graw S, Camerota M, Carter BS, Helderman J, Hofheimer JA, 2021. NEOage clocks—epigenetic clocks to estimate post-menstrual and postnatal age in preterm infants. Aging 13(20):23527–44 34655469 10.18632/aging.203637 PMC8580352

[53] Grodstein F, Lemos B, Yu L, Iatrou A, De Jager PL, Bennett DA. 2021. Characteristics of epigenetic clocks across blood and brain tissue in older women and men. Front. Neurosci 14:555307 33488342 10.3389/fnins.2020.555307 PMC7817909

[54] Guvatova ZG, Kobelyatskaya AA, Pudova EA, Tarasova IV, Kudryavtseva AV, 2023. Decelerated epigenetic aging in long livers. Int. J. Mol. Sci 24(23):16867 38069189 10.3390/ijms242316867 PMC10707056

[55] Hamlat EJ, Prather AA, Horvath S, Belsky J, Epel ES. 2021. Early life adversity, pubertal timing, and epigenetic age acceleration in adulthood. Dev. Psychobiol 63(5):890–902 33423276 10.1002/dev.22085 PMC8271092

[56] Hannum G, Guinney J, Zhao L, Zhang L, Hughes G, 2013. Genome-wide methylation profiles reveal quantitative views of human aging rates. Mol. Cell 49(2):359–67 23177740 10.1016/j.molcel.2012.10.016 PMC3780611

[57] Harvanek ZM, Fogelman N, Xu K, Sinha R. 2021. Psychological and biological resilience modulates the effects of stress on epigenetic aging. Transl. Psychiatry 11:601 34839356 10.1038/s41398-021-01735-7 PMC8627511

[58] Harvanek ZM, Kudinova AY, Wong SA, Xu K, Brick L, 2024. Childhood adversity, accelerated GrimAge, and associated health consequences. J. Behav. Med 47:913–26 38762606 10.1007/s10865-024-00496-0 PMC11365810

[59] Hillary RF, Stevenson AJ, Cox SR, McCartney DL, Harris SE, 2021. An epigenetic predictor of death captures multi-modal measures of brain health. Mol. Psychiatry 26:3806–16 31796892 10.1038/s41380-019-0616-9 PMC8550950

[60] Holloway TD, Harvanek ZM, Xu K, Gordon DM, Sinha R. 2023. Greater stress and trauma mediate race-related differences in epigenetic age between Black and White young adults in a community sample. Neurobiol. Stress 26:100557 37501940 10.1016/j.ynstr.2023.100557 PMC10369475

[61] Horvath S. 2013. DNA methylation age of human tissues and cell types. Genome Biol. 14(10):R115 24138928 10.1186/gb-2013-14-10-r115 PMC4015143

[62] Horvath S, Gurven M, Levine ME, Trumble BC, Kaplan H, 2016. An epigenetic clock analysis of race/ethnicity, sex, and coronary heart disease. Genome Biol. 17(1):171 27511193 10.1186/s13059-016-1030-0 PMC4980791

[63] Horvath S, Lu AT, Haghani A, Zoller JA, Li CZ, 2022. DNA methylation clocks for dogs and humans. PNAS 119(21):e2120887119 35580182 10.1073/pnas.2120887119 PMC9173771

[64] Horvath S, Mah V, Lu AT, Woo JS, Choi O-W, 2015. The cerebellum ages slowly according to the epigenetic clock. Aging 7(5):294–305 26000617 10.18632/aging.100742 PMC4468311

[65] Horvath S, Oshima J, Martin GM, Lu AT, Quach A, 2018. Epigenetic clock for skin and blood cells applied to Hutchinson Gilford Progeria Syndrome and *ex vivo* studies. Aging 10(7):1758–75 30048243 10.18632/aging.101508 PMC6075434

[66] Horvath S, Pirazzini C, Bacalini MG, Gentilini D, Di Blasio AM, 2015. Decreased epigenetic age of PBMCs from Italian semi-supercentenarians and their offspring. Aging 7(12):1159–70 26678252 10.18632/aging.100861 PMC4712339

[67] Horvath S, Raj K. 2018. DNA methylation-based biomarkers and the epigenetic clock theory of ageing. Nat. Rev. Genet 19:371–84 29643443 10.1038/s41576-018-0004-3

[68] Horvath S, Singh K, Raj K, Khairnar S, Sanghavi A, 2023. Reversal of biological age in multiple rat organs by young porcine plasma fraction. Geroscience 46:367–94 37875652 10.1007/s11357-023-00980-6 PMC10828479

[69] Jiang EX, Domingo-Relloso A, Abuawad A, Haack K, Tellez-Plaza M, 2023. Arsenic exposure and epigenetic aging: the association with cardiovascular disease and all-cause mortality in the Strong Heart Study. Environ. Health Perspect 131(12):127016 38133959 10.1289/EHP11981 PMC10743589

[70] Johnson AA, Torosin NS, Shokhirev MN, Cuellar TL. 2022. A set of common buccal CpGs that predict epigenetic age and associate with lifespan-regulating genes. iScience 25(11):105304 36304118 10.1016/j.isci.2022.105304 PMC9593711

[71] Joshi D, van Lenthe FJ, Huisman M, Sund ER, Krokstad S, 2024. Association of neighborhood deprivation and depressive symptoms with epigenetic age acceleration: evidence from the Canadian Longitudinal Study on Aging. J. Gerontol. A 79(2):glad118 10.1093/gerona/glad118 PMC10809038 37279588

[72] Kabacik S, Lowe D, Fransen L, Leonard M, Ang S-L, 2022. The relationship between epigenetic age and the hallmarks of aging in human cells. Nat. Aging 2(6):484–93 37034474 10.1038/s43587-022-00220-0 PMC10077971

[73] Katrinli S, Stevens J, Wani AH, Lori A, Kilaru V, 2020. Evaluating the impact of trauma and PTSD on epigenetic prediction of lifespan and neural integrity. Neuropsychopharmacology 45(10):1609–16 32380512 10.1038/s41386-020-0700-5 PMC7421899

[74] Kim K, Yaffe K, Rehkopf DH, Zheng Y, Nannini DR, 2023. Association of adverse childhood experiences with accelerated epigenetic aging in midlife. JAMA Netw. Open 6(6):e2317987 37306997 10.1001/jamanetworkopen.2023.17987 PMC10261996

[75] Kim K, Zheng Y, Joyce BT, Jiang H, Greenland P, 2022. Relative contributions of six lifestyle- and health-related exposures to epigenetic aging: the Coronary Artery Risk Development in Young Adults (CARDIA) Study. Clin. Epigenet 14(1):85 10.1186/s13148-022-01304-9 PMC9264709 35799271

[76] Kim Y, Huan T, Joehanes R, McKeown NM, Horvath S, 2022. Higher diet quality relates to decelerated epigenetic aging. Am. J. Clin. Nutr 115(1):163–70 34134146 10.1093/ajcn/nqab201 PMC8755029

[77] Klopack ET, Carroll JE, Cole SW, Seeman TE, Crimmins EM. 2022. Lifetime exposure to smoking, epigenetic aging, and morbidity and mortality in older adults. Clin. Epigenet 14(1):72 10.1186/s13148-022-01286-8 PMC9148451 35643537

[78] Knight AK, Craig JM, Theda C, Bækvad-Hansen M, Bybjerg-Grauholm J, 2016. An epigenetic clock for gestational age at birth based on blood methylation data. Genome Biol. 17(1):206 27717399 10.1186/s13059-016-1068-z PMC5054584

[79] Koenigsberg SH, Chang C-J, Ish J, Xu Z, Kresovich JK, 2023. Air pollution and epigenetic aging among Black and White women in the US. Environ. Int 181:108270 37890265 10.1016/j.envint.2023.108270 PMC10872847

[80] Komaki S, Nagata M, Arai E, Otomo R, Ono K, 2023. Epigenetic profile of Japanese supercentenarians: a cross-sectional study. Lancet Healthy Longev. 4(2):e83–90 36738748 10.1016/S2666-7568(23)00002-8

[81] Korous KM, Surachman A, Rogers CR, Cuevas AG. 2023. Parental education and epigenetic aging in middle-aged and older adults in the United States: a life course perspective. Soc. Sci. Med 333:116173 37595421 10.1016/j.socscimed.2023.116173 PMC10530379

[82] Kresovich JK, Park Y-MM, Keller JA, Sandler DP, Taylor JA. 2022. Healthy eating patterns and epigenetic measures of biological age. Am. J. Clin. Nutr 115(1):171–79 34637497 10.1093/ajcn/nqab307 PMC8754996

[83] Kuo C-L, Chen Z, Liu P, Pilling LC, Atkins JL. 2023. Proteomic aging clock (PAC) predicts age-related outcomes in middle-aged and older adults. Aging Cell 23:e14195 10.1111/acel.14195 PMC11320350 38747160

[84] Kuo P-L, Moore AZ, Tanaka T, Belsky DW, Tzu-Hui Lu A, 2024. Longitudinal changes in epigenetic clocks predict survival in the InCHIANTI cohort. medRxiv. 10.1101/2024.09.13.24313620 PMC13004684 41826710

[85] Kusters CDJ, Klopack ET, Crimmins EM, Seeman T, Cole S, Carroll JE. 2024. Short sleep and insomnia are associated with accelerated epigenetic age. Psychosom. Med 86:453–62 37594243 10.1097/PSY.0000000000001243 PMC10879461

[86] Kusters CDJ, Paul KC, Lu AT, Ferruci L, Ritz BR, 2024. Higher testosterone and testosterone/estradiol ratio in men are associated with decreased Pheno/GrimAge and DNA methylation based PAI1. Geroscience 46:1053–69 37369886 10.1007/s11357-023-00832-3 PMC10828310

[87] Lawrence KG, Kresovich JK, O’Brien KM, Hoang TT, Xu Z, 2020. Association of neighborhood deprivation with epigenetic aging using 4 clock metrics. JAMA Netw. Open 3(11):e2024329 33146735 10.1001/jamanetworkopen.2020.24329 PMC7643028

[88] Lee Y, Choufani S, Weksberg R, Wilson SL, Yuan V, 2019. Placental epigenetic clocks: estimating gestational age using placental DNA methylation levels. Aging 11(12):4238–53 31235674 10.18632/aging.102049 PMC6628997

[89] Lei M-K, Berg MT, Simons RL, Beach SRH. 2022. Neighborhood structural disadvantage and biological aging in a sample of Black middle age and young adults. Soc. Sci. Med 293:114654 34923353 10.1016/j.socscimed.2021.114654 PMC8810597

[90] Levine ME, Lu AT, Chen BH, Hernandez DG, Singleton AB, 2016. Menopause accelerates biological aging. PNAS 113(33):9327–32 27457926 10.1073/pnas.1604558113 PMC4995944

[91] Levine ME, Lu AT, Quach A, Chen BH, Assimes TL, 2018. An epigenetic biomarker of aging for lifespan and healthspan. Aging 10(4):573–91 29676998 10.18632/aging.101414 PMC5940111

[92] Li DL, Hodge AM, Cribb L, Southey MC, Giles GG, 2024. Body size, diet quality, and epigenetic aging: cross-sectional and longitudinal analyses. J. Gerontol. A 79:glae026 10.1093/gerona/glae026 PMC10953795 38267386

[93] Li M, Bao L, Zhu P, Wang S. 2022. Effect of metformin on the epigenetic age of peripheral blood in patients with diabetes mellitus. Front. Genet 13:955835 36226195 10.3389/fgene.2022.955835 PMC9548538

[94] Lim S, Nzegwu D, Wright ML. 2022. The impact of psychosocial stress from life trauma and racial discrimination on epigenetic aging—a systematic review. Biol. Res. Nurs 24(2):202–15 35102751 10.1177/10998004211060561 PMC9096197

[95] Lin W-Y. 2022. Genome-wide association study for four measures of epigenetic age acceleration and two epigenetic surrogate markers using DNA methylation data from Taiwan Biobank. Hum. Mol. Genet 31(11):1860–70 34962275 10.1093/hmg/ddab369

[96] Liu Y, Li C. 2024. Hormone therapy and biological aging in postmenopausal women. JAMA Netw. Open 7(8):e2430839 39207753 10.1001/jamanetworkopen.2024.30839 PMC11362863

[97] Liu Z, Chen BH, Assimes TL, Ferrucci L, Horvath S, Levine ME. 2019. The role of epigenetic aging in education and racial/ethnic mortality disparities among older U.S. women. Psychoneuroendocrinology 104:18–24 30784901 10.1016/j.psyneuen.2019.01.028 PMC6555423

[98] Liu Z, Leung D, Thrush K, Zhao W, Ratliff S, 2020. Underlying features of epigenetic aging clocks in vivo and in vitro. Aging Cell 19(10):e13229 32930491 10.1111/acel.13229 PMC7576259

[99] López-Otín C, Blasco MA, Partridge L, Serrano M, Kroemer G. 2013. The hallmarks of aging. Cell 153(6):1194–217 23746838 10.1016/j.cell.2013.05.039 PMC3836174

[100] López-Otín C, Blasco MA, Partridge L, Serrano M, Kroemer G. 2023. Hallmarks of aging: an expanding universe. Cell 186(2):243–78 36599349 10.1016/j.cell.2022.11.001

[101] Lu AT, Binder AM, Zhang J, Yan Q, Reiner AP, 2022. DNA methylation GrimAge version 2. Aging 14(23):9484–549 36516495 10.18632/aging.204434 PMC9792204

[102] Lu AT, Fei Z, Haghani A, Robeck TR, Zoller JA, 2023. Universal DNA methylation age across mammalian tissues. Nat. Aging 3(9):1144–66 37563227 10.1038/s43587-023-00462-6 PMC10501909

[103] Lu AT, Quach A, Wilson JG, Reiner AP, Aviv A, 2019. DNA methylation GrimAge strongly predicts lifespan and healthspan. Aging 11(2):303–27 30669119 10.18632/aging.101684 PMC6366976

[104] Lyons CE, Razzoli M, Bartolomucci A. 2023. The impact of life stress on hallmarks of aging and accelerated senescence: connections in sickness and in health. Neurosci. Biobehav. Rev 153:105359 37586578 10.1016/j.neubiorev.2023.105359 PMC10592082

[105] Malhotra N, Malhotra J, Bora NM, Bora R, Malhotra K. 2014. Fetal origin of adult disease. Donald Sch. J. Ultrasound Obstet. Gynecol 8(2):164–77

[106] Maunakea AK, Phankitnirundorn K, Peres R, Dye C, Juarez R, 2024. Socioeconomic status, lifestyle, and DNA methylation age among racially and ethnically diverse adults: NIMHD social epigenomics program. JAMA Netw. Open 7(7):e2421889 39073814 10.1001/jamanetworkopen.2024.21889 PMC11287425

[107] Mayne BT, Leemaqz SY, Smith AK, Breen J, Roberts CT, Bianco-Miotto T. 2017. Accelerated placental aging in early onset preeclampsia pregnancies identified by DNA methylation. Epigenomics 9(3):279–89 27894195 10.2217/epi-2016-0103 PMC6040051

[108] Mboning L, Rubbi L, Thompson M, Bouchard L-S, Pellegrini M. 2024. BayesAge: a maximum likelihood algorithm to predict epigenetic age. Front. Bioinform 4:1329144 38638123 10.3389/fbinf.2024.1329144 PMC11024280

[109] McCrory C, Fiorito G, Hernandez B, Polidoro S, O’Halloran AM, 2021. GrimAge outperforms other epigenetic clocks in the prediction of age-related clinical phenotypes and all-cause mortality. J. Gerontol. A 76(5):741–49 10.1093/gerona/glaa286 PMC8087266 33211845

[110] McCrory C, Fiorito G, O’Halloran AM, Polidoro S, Vineis P, Kenny RA. 2022. Early life adversity and age acceleration at mid-life and older ages indexed using the next-generation GrimAge and Pace of Aging epigenetic clocks. Psychoneuroendocrinology 137:105643 34999481 10.1016/j.psyneuen.2021.105643

[111] McEwen LM, O’Donnell KJ, McGill MG, Edgar RD, Jones MJ, 2020. The PedBE clock accurately estimates DNA methylation age in pediatric buccal cells. PNAS 117(38):23329–35 31611402 10.1073/pnas.1820843116 PMC7519312

[112] Mehta D, Bruenig D, Lawford B, Harvey W, Carrillo-Roa T, 2018. Accelerated DNA methylation aging and increased resilience in veterans: the biological cost for soldiering on. Neurobiol. Stress 8:112–19 29888306 10.1016/j.ynstr.2018.04.001 PMC5991315

[113] Mendy A, Mersha TB. 2024. Epigenetic age acceleration and mortality prediction in U.S. adults. medRxiv. 10.1101/2024.08.21.24312373 PMC12397028 40095187

[114] Moffitt TE. 2024. Progress of research on the PACE of aging and literature review. DunedinPACE. https://moffittcaspi.trinity.duke.edu/sites/moffittcaspi.trinity.duke.edu/files/documents/DunedinPACE%20validation%20literature%20SUMMARY%203Jan2024.pdf

[115] Moqri M, Cipriano A, Nachun D, Murty T, de Sena Brandine G, 2022. PRC2 clock: a universal epigenetic biomarker of aging and rejuvenation. bioRxiv. 10.1101/2022.06.03.494609 PMC11250797 39009581

[116] Nielsen JL, Bakula D, Scheibye-Knudsen M. 2022. Clinical trials targeting aging. Front. Aging 3. 10.3389/fragi.2022.820215 PMC9261384 35821843

[117] Noroozi R, Rudnicka J, Pisarek A, Wysocka B, Masny A, 2024. Analysis of epigenetic clocks links yoga, sleep, education, reduced meat intake, coffee, and a SOCS2 gene variant to slower epigenetic aging. Geroscience 46(2):2583–604 38103096 10.1007/s11357-023-01029-4 PMC10828238

[118] Nwanaji-Enwerem JC, Cardenas A, Gao X, Wang C, Vokonas P, 2023. Psychological stress and epigenetic aging in older men: the VA Normative Aging Study. Transl. Med. Aging 7:66–74 37576443 10.1016/j.tma.2023.06.003 PMC10416788

[119] Oblak L, van der Zaag J, Higgins-Chen AT, Levine ME, Boks MP. 2021. A systematic review of biological, social and environmental factors associated with epigenetic clock acceleration. Ageing Res. Rev 69:101348 33930583 10.1016/j.arr.2021.101348

[120] Oh HS-H, Rutledge J, Nachun D, Pálovics R, Abiose O, 2023. Organ aging signatures in the plasma proteome track health and disease. Nature 624(7990):164–72 38057571 10.1038/s41586-023-06802-1 PMC10700136

[121] Palma-Gudiel H, Fañanás L, Horvath S, Zannas AS. 2020. Psychosocial stress and epigenetic aging. Int. Rev. Neurobiol 150:107–28 32204828 10.1016/bs.irn.2019.10.020

[122] Phang M, Ross J, Raythatha JH, Dissanayake HU, McMullan RL, 2020. Epigenetic aging in newborns: role of maternal diet. Am. J. Clin. Nutr 111(3):555–61 31942922 10.1093/ajcn/nqz326

[123] Phyo AZZ, Fransquet PD, Wrigglesworth J, Woods RL, Espinoza SE, Ryan J. 2024. Sex differences in biological aging and the association with clinical measures in older adults. Geroscience 46(2):1775–88 37747619 10.1007/s11357-023-00941-z PMC10828143

[124] Prosz A, Pipek O, Börcsök J, Palla G, Szallasi Z, 2024. Biologically informed deep learning for explainable epigenetic clocks. Sci. Rep 14(1):1306 38225268 10.1038/s41598-023-50495-5 PMC10789766

[125] Protsenko E, Wolkowitz OM, Yaffe K. 2023. Associations of stress and stress-related psychiatric disorders with GrimAge acceleration: review and suggestions for future work. Transl. Psychiatry 13:142 37130894 10.1038/s41398-023-02360-2 PMC10154294

[126] Quach A, Levine ME, Tanaka T, Lu AT, Chen BH, 2017. Epigenetic clock analysis of diet, exercise, education, and lifestyle factors. Aging 9(2):419–37 28198702 10.18632/aging.101168 PMC5361673

[127] Raffington L, Belsky DW. 2022. Integrating DNA methylation measures of biological aging into social determinants of health research. Curr. Environ. Health Rep 9(2):196–210 35181865 10.1007/s40572-022-00338-8

[128] Raffington L, Belsky DW, Kothari M, Malanchini M, Tucker-Drob EM, Harden KP. 2021. Socioeconomic disadvantage and the pace of biological aging in children. Pediatrics 147(6):e2020024406 34001641 10.1542/peds.2020-024406 PMC8785753

[129] Saeed H, Wu J, Tesfaye M, Grantz KL, Tekola-Ayele F. 2024. Placental accelerated aging in antenatal depression. Am. J. Obstet. Gynecol. MFM 6(1):101237 38012987 10.1016/j.ajogmf.2023.101237 PMC10843762

[130] Sehgal R, Markov Y, Qin C, Meer M, Hadley C, 2023. Systems age: a single blood methylation test to quantify aging heterogeneity across 11 physiological systems. bioRxiv. 10.1101/2023.07.13.548904 PMC13222069 40954326

[131] Shen B, Mode NA, Noren Hooten N, Pacheco NL, Ezike N, 2023. Association of race and poverty status with DNA methylation-based age. JAMA Netw. Open 6(4):E236340 37027157 10.1001/jamanetworkopen.2023.6340 PMC10082406

[132] Shireby GL, Davies JP, Francis PT, Burrage J, Walker EM, 2020. Recalibrating the epigenetic clock: implications for assessing biological age in the human cortex. Brain 143(12):3763–75 33300551 10.1093/brain/awaa334 PMC7805794

[133] Shokhirev MN, Torosin NS, Kramer DJ, Johnson AA, Cuellar TL. 2024. CheekAge: a next-generation buccal epigenetic aging clock associated with lifestyle and health. Geroscience 46(3):3429–43 38441802 10.1007/s11357-024-01094-3 PMC11009193

[134] Short AK, Weber R, Kamei N, Wilcox Thai C, Arora H, 2024. Individual longitudinal changes in DNA-methylome identify signatures of early-life adversity and correlate with later outcome. Neurobiol. Stress 31:100652 38962694 10.1016/j.ynstr.2024.100652 PMC11219970

[135] Sillanpää E, Heikkinen A, Kankaanpää A, Paavilainen A, Kujala UM, 2021. Blood and skeletal muscle ageing determined by epigenetic clocks and their associations with physical activity and functioning. Clin. Epigenet 13(1):110 10.1186/s13148-021-01094-6 PMC8127311 34001218

[136] Simons RL, Lei M-K, Klopach E, Berg M, Zhang Y, Beach SSR. 2021. Re(setting) epigenetic clocks: an important avenue whereby social conditions become biologically embedded across the life course. J. Health Soc. Behav 62(3):436–53 34528488 10.1177/00221465211009309

[137] Simons RL, Lei M-K, Klopack E, Beach SRH, Gibbons FX, Philibert RA. 2021. The effects of social adversity, discrimination, and health risk behaviors on the accelerated aging of African Americans: further support for the weathering hypothesis. Soc. Sci. Med 282:113169 32690336 10.1016/j.socscimed.2020.113169 PMC7790841

[138] Skinner HG, Palma-Gudiel H, Stewart JD, Love S-A, Bhatti P, 2024. Stressful life events, social support, and epigenetic aging in the Women’s Health Initiative. J. Am. Geriatr. Soc 72(2):349–60 38149693 10.1111/jgs.18726 PMC10922473

[139] Song AY, Feinberg JI, Bakulski KM, Croen LA, Fallin MD, 2022. Prenatal exposure to ambient air pollution and epigenetic aging at birth in newborns. Front. Genet 13:929416 35836579 10.3389/fgene.2022.929416 PMC9274082

[140] Sosnowski DW, Rojo-Wissar DM, Peng G, Parade SH, Sharkey K, 2024. Maternal childhood adversity and infant epigenetic aging: moderation by restless sleep during pregnancy. Dev. Psychobiol 66(2):e22464 38601952 10.1002/dev.22464 PMC11003750

[141] Stevenson AJ, McCartney DL, Gadd DA, Shireby G, Hillary RF, 2022. A comparison of blood and brain-derived ageing and inflammation-related DNA methylation signatures and their association with microglial burdens. Eur. J. Neurosci 56(9):5637–49 35362642 10.1111/ejn.15661 PMC9525452

[142] Sullivan ADW, Bozack AK, Cardenas A, Comer JS, Bagner DM, 2023. Parenting practices may buffer the impact of adversity on epigenetic age acceleration among young children with developmental delays. Psychol. Sci 34(10):1173–85 37733001 10.1177/09567976231194221 PMC10626625

[143] Tamargo JA, Cruz-Almeida Y. 2024. Food insecurity and epigenetic aging in middle-aged and older adults. Soc. Sci. Med 350:116949 38723585 10.1016/j.socscimed.2024.116949 PMC11848750

[144] Tanaka T, Basisty N, Fantoni G, Candia J, Moore AZ, 2020. Plasma proteomic biomarker signature of age predicts health and life span. eLife 9:e61073 33210602 10.7554/eLife.61073 PMC7723412

[145] Tangili M, Slettenhaar AJ, Sudyka J, Dugdale HL, Pen I, 2023. DNA methylation markers of age(ing) in non-model animals. Mol. Ecol 32(17):4725–41 37401200 10.1111/mec.17065

[146] Thomas A, Ryan CP, Caspi A, Liu Z, Moffitt TE, 2024. Diet, pace of biological aging, and risk of dementia in the Framingham Heart Study. Ann. Neurol 95:1069–79 38407506 10.1002/ana.26900 PMC11102315

[147] Voisin S, Harvey NR, Haupt LM, Griffiths LR, Ashton KJ, 2020. An epigenetic clock for human skeletal muscle. J. Cachexia Sarcopenia Muscle 11(4):887–98 32067420 10.1002/jcsm.12556 PMC7432573

[148] Wang M, Li Y, Lai M, Nannini DR, Hou L, 2023. Alcohol consumption and epigenetic age acceleration across human adulthood. Aging 15(20):10938–71 37889500 10.18632/aging.205153 PMC10637803

[149] Wang S, Li W, Li S, Tu H, Jia J, 2023. Association between plant-based dietary pattern and biological aging trajectory in a large prospective cohort. BMC Med. 21(1):310 37592257 10.1186/s12916-023-02974-9 PMC10433678

[150] Warner B, Ratner E, Datta A, Lendasse A. 2024. A systematic review of phenotypic and epigenetic clocks used for aging and mortality quantification in humans. Aging 16:12414–27 39215995 10.18632/aging.206098 PMC11424583

[151] Watkins SH, Testa C, Chen JT, De Vivo I, Simpkin AJ, 2023. Epigenetic clocks and research implications of the lack of data on whom they have been developed: a review of reported and missing sociodemographic characteristics. Environ. Epigenet 9:dvad005 37564905 10.1093/eep/dvad005 PMC10411856

[152] Waziry R, Ryan CP, Corcoran DL, Huffman KM, Kobor MS, 2023. Effect of long-term caloric restriction on DNA methylation measures of biological aging in healthy adults from the CALERIE trial. Nat. Aging 3(3):248–57 37118425 10.1038/s43587-022-00357-y PMC10148951

[153] Weidner CI, Lin Q, Koch CM, Eisele L, Beier F, 2014. Aging of blood can be tracked by DNA methylation changes at just three CpG sites. Genome Biol. 15(2):R24 24490752 10.1186/gb-2014-15-2-r24 PMC4053864

[154] White AJ, Kresovich JK, Keller JP, Xu Z, Kaufman JD, 2019. Air pollution, particulate matter composition and methylation-based biologic age. Environ. Int 132:105071 31387022 10.1016/j.envint.2019.105071 PMC6754788

[155] Wu X, Huang Q, Javed R, Zhong J, Gao H, Liang H. 2019. Effect of tobacco smoking on the epigenetic age of human respiratory organs. Clin. Epigenet 11(1):183 10.1186/s13148-019-0777-z PMC6894291 31801625

[156] Yang R, Wu GWY, Verhoeven JE, Gautam A, Reus VI, 2021. A DNA methylation clock associated with age-related illnesses and mortality is accelerated in men with combat PTSD. Mol. Psychiatry 26(9):4999–5009 32382136 10.1038/s41380-020-0755-z

[157] Yang Y, Lu X, Liu N, Ma S, Zhang H, 2024. Metformin decelerates aging clock in male monkeys. Cell 187:6358–78.e29 39270656 10.1016/j.cell.2024.08.021

[158] Yaskolka Meir A, Keller M, Hoffmann A, Rinott E, Tsaban G, 2023. The effect of polyphenols on DNA methylation-assessed biological age attenuation: the DIRECT PLUS randomized controlled trial. BMC Med. 21(1):364 37743489 10.1186/s12916-023-03067-3 PMC10519069

[159] Ying K, Liu H, Tarkhov AE, Sadler MC, Lu AT, 2024. Causality-enriched epigenetic age uncouples damage and adaptation. Nat. Aging 4(2):231–46 38243142 10.1038/s43587-023-00557-0 PMC11070280

[160] Yusipov I, Kalyakulina A, Franceschi C, Ivanchenko M. 2024. Map of epigenetic age acceleration: a worldwide meta-analysis. bioRxiv. 10.1101/2024.03.17.585398 39002646

[161] Zannas AS, Arloth J, Carrillo-Roa T, Iurato S, Röh S, 2015. Lifetime stress accelerates epigenetic aging in an urban, African American cohort: relevance of glucocorticoid signaling. Genome Biol. 16(1):266 26673150 10.1186/s13059-015-0828-5 PMC4699359

[162] Zhang A, Zhang Y, Meng Y, Ji Q, Ye M, 2024. Associations between psychological resilience and epigenetic clocks in the health and retirement study. Geroscience 46(1):961–68 37707649 10.1007/s11357-023-00940-0 PMC10828333

[163] Zhang Q, Vallerga CL, Walker RM, Lin T, Henders AK, 2019. Improved precision of epigenetic clock estimates across tissues and its implication for biological ageing. Genome Med. 11(1):54 31443728 10.1186/s13073-019-0667-1 PMC6708158

[164] Zhao W, Ammous F, Ratliff S, Liu J, Yu M, 2019. Education and lifestyle factors are associated with DNA methylation clocks in older African Americans. Int. J. Environ. Res. Public Health 16(17):3141 31466396 10.3390/ijerph16173141 PMC6747433

